# Cerebral Ischemia after Stenting of Coarctation of the Aorta

**DOI:** 10.1155/2021/8868312

**Published:** 2021-02-20

**Authors:** D. Doughmi, S. Benlamkaddem, M. A. Berdai, S. Atmani, M. Harandou

**Affiliations:** ^1^Department of Pediatric Anesthesia and Intensive Care, Hassan II Academic Hospital, Fez, Morocco; ^2^Department of Pediatric Cardiology and Pediatric Cardiac Catheterization, Hassan II Academic Hospital, Fez, Morocco

## Abstract

Percutaneous stenting angioplasty of native coarctation of the aorta is considered a low-risk procedure with high success rate. The incidence of cerebral complications, especially ischemic complications, is very low. We report a case of a 15-year-old boy who underwent a percutaneous stenting angioplasty for a coarctation of the aorta and developed a cerebral infraction 4 hours after the procedure.

## 1. Introduction

Coarctation of the aorta is a relatively common defect. It may occur as an isolated defect or in association with a variety of other lesions, most commonly bicuspid aortic valve. The endovascular stent angioplasty in the treatment of coarctation holds multiple theoretic advantages; there is no requirement for an intimal tear with a stent, so the risk for aortic wall injury is lower [1]. The major reported complications of the angioplasty are death, rupture of the aorta, recoarctation, aneurysm formation at the site of repair, cerebrovascular accident, and femoral artery thrombosis [2]. The incidence of cerebral ischemic complications is very low, and the physiopathology is still unknown; it can be a result of an embolism, a variation in the cerebral blood flow, or a variation in the anatomy.

## 2. Case Report

A 15-year-old patient with no medical history was admitted to the emergency department for severe myalgia of the lower extremities for 6 months. The blood pressure of the upper extremities was 180/100 mmHg and that of the lower extremities was 80/50 mmHg. Cardiovascular examination revealed pansystolic murmur in the aortic valve. An echocardiography showed an aortic isthmus coarctation of 3 mm, just after the origin of the left subclavian artery. The diameter of the stenotic area was very tight with a peak velocity of 4.3 m/s. The diameter of poststenotic area was 25 mm.

**Figure 1:**
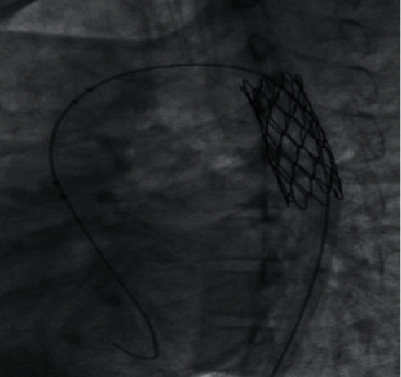
Aortography showing the stent in place.

Interventional transcatheter stenting was performed under general anesthesia; the induction was performed by administering 100 *μ*g of fentanyl, 100 mg of propofol, and 30 mg of rocuronium; the anesthesia was maintained by sevoflurane and titration of fentanyl and propofol. The aim of our hemodynamic management was to maintain a median blood pressure of 80 mmHg and systolic blood pressure of 110 mmHg for cerebral perfusion. The initial aortography by femoral artery was impossible despite meticulous injection from the descendant aorta. Our decision was to perform the aortography by the radial artery; it showed a very tight coarctation with two aneurysms in the descending aorta. The coarctation was crossed by balloon 4 mm mounted over 0.35 Fr guide, and the angioplasty was performed by using a drug-eluting 39/16 mm stent after heparinotherapy by femoral artery (Figure 1). The treatment outcome was good, with significant reduction in pressure gradient across the stent.

**Figure 2:**
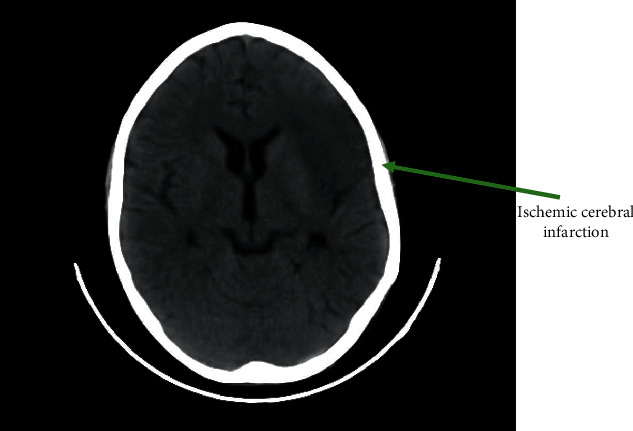
A cerebral CT scan revealing a hypodense lesion on the left frontal lobe indicating a recent ischemic cerebral infarction.

**Figure 3:**
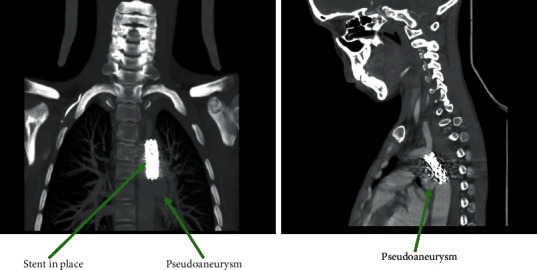
The thoracic angio-CT scan revealing a good opacification of the ascending aorta and the aortic arch with stent in place, with two sacciform pseudoaneurysms in the descending aorta just below the stent.

Immediately after the angioplasty, the patient presented a delayed awakening that was explained initially by the anesthetic agents used. After 4 hours, the patient regained consciousness but with a GCS of 11 and a right hemiplegia. A cerebral CT scan revealed a hypodense lesion on the left frontal lobe indicating a recent ischemic cerebral infarction (Figure 2). The angio-CT scan showed an opacification defect of the distal M1 branch and left M2 extended on 10 mm. There was a good opacification of the ascending aorta and the aortic arch with stent in place. We found two sacciform pseudoaneurysms in the descending aorta just below the stent; one is anterior measuring 25 mm with a collar of 8.5 mm and the other is lateral measuring 20.5 mm with a collar of 6 mm (Figure 3).

The patient was admitted in the intensive care unit; he received motor kinesitherapy with a dual antiplatelet therapy based on aspirin (70 mg per day) and clopidogrel (75 mg per day). The outcome was favorable; he regained his motor functions in his right side.

## 3. Discussion

Coarctation of the aorta is a congenital narrowing of the upper descending aorta. The approximate incidence is 4% in live-born children with congenital heart disease [3]. Although it can be easily detected by femoral pulse palpation, it is often undiagnosed until late childhood.

The lesion was first eloquently described by Giovanni Morgagni, and the first surgical intervention was performed by Crafoord in 1944 [4, 5]. Balloon angioplasty of the aorta was first described by Singer in 1982 and by Lock et al. in 1983 [6, 7].

The incidence of cerebral complications following balloon angioplasty or stent placement is low [8]. Cerebral hemorrhage in patients with coarctation of the aorta has been attributed to the aneurysms of the circle of Willis and the presence of systemic hypertension. Opio et al. [9] reported the case of a 14-year-old girl with no medical history who was admitted for loss of consciousness associated with right hemiplegia and facial palsy. Nonenhanced brain CT scan showed a well-defined homogeneously hyperdense mass lesion in the left frontotemporal periventricular region; a diagnosis of acute intracerebral hemorrhage was made. The echocardiography showed left ventricular hypertrophy, aortic stenosis, and a small degree of aortic incompetence. Daghero et al. [10] reported a case of an acute hemorrhagic stroke in a 14-year-old as a sign of coarctation of abdominal aorta; the magnetic resonance imaging of the brain revealed subarachnoid hemorrhage secondary to an aneurysm of the left posterior communicating artery.

The incidence of cerebral infraction secondary to angioplasty repair of coarctation of aorta is rare [11]. It was the first case in our department. The study conducted by Lefort et al. [8] about the outcome of balloon angioplasty for recurrent aortic coarctation in patients aged less than 1 year showed that only one patient among 20 developed a transient ischemic stroke with complete recovery at discharge.

The physiopathology is still unknown. Dehghan et al. [12] reported a case of a 32-year-old man who developed after a transcatheter aortic stenting symptoms of transient cerebral ischemia secondary to a complete occlusion of the origin of aberrant right subclavian artery (ARSA) which was originating at the site of coarctation. Color Doppler sonography revealed a retrograde flow through the right-side vertebral artery, indicating the subclavian steal syndrome. In Gogou et al.'s case [13], the cerebral ischemia post balloon angioplasty is most likely due to variations in cerebral blood flow. These types of patients are chronically exposed to high cerebral blood pressure, and the reduction of blood supply could result in ischemic episodes.

In our case, the cerebral ischemia is due to embolism; the angio-CT scan showed an opacification defect of the distal M1 branch and left M2. Ussia et al. [14] have reported a case of paraplegia following balloon angioplasty of aortic coarctation in an infant, most likely due to embolism.

Risk factors for stroke after thoracic endovascular aortic repair (TEVAR) according to Ullery et al. include previous stroke, a more proximal extent of repair, high-grade aortic arch atheroma, intraoperative hypotension, and chronic renal insufficiency [15].

Anisha et al. conducted a study to investigate cerebral embolization following thoracic endovascular aortic repair (TEVAR) [16]. They included 52 patients undergoing elective or emergency TEVAR. Only four patients developed clinical stroke. Two patients made a complete recovery following a period of rehabilitation.

The antiplatelet therapy is a key component of the management of noncardioembolic ischemic stroke and transient ischemic attack (TIA). In acute cerebral infraction, the incidence of recurrent ischemic strokes in patients receiving dual antiplatelet therapy (aspirin with clopidogrel) was less noted than in patients receiving aspirin in monotherapy [17]. For long-term prevention of recurrent vascular events in patients with a history of ischemic stroke or TIA, an antiplatelet therapy must be used. Aspirin in monotherapy has a strong track record in acute and chronic cerebrovascular ischemia but clearly does not prevent from all recurrent events, and dual antiplatelet therapy exposes the patient for hemorrhagic complications [18].

According to Godart, drug-eluting stents did not show any superiority over bare stents in terms of efficacy, aortic diameter expansion, and gradient reduction, but they are preferred in situations such as subatretic coarctation, native coarctation associated with patent ductus arteriosus, (re)coarctation combined with aneurysm, and recurrent coarctation after PTFE patch [19].

The European Society of Cardiology recommends the use of dual antiplatelet therapy for 6 to 12 months or for at least 12 months after drug-eluting stent implantation unless patients are at high risk for bleeding [20]. In our case, we used dual antiplatelet therapy immediately after the endovascular procedure and it was given for at least 6 months; after that, aspirin in monotherapy was continued for life.

## 4. Conclusion

Stent implantation is a good alternative to surgery for the treatment of coarctation of the aorta. It is associated with a low-residual gradient and a low rate of restenosis, both immediately and at follow-up. The incidence for a stroke after endovascular procedure is very low, and the physiopathology is still unknown; it can be a result of an embolism, a variation in the cerebral blood flow, or a variation in the anatomy. Antiplatelet therapy is a key component of the management of cerebral infraction.

## Data Availability

The data used to support the study can be available upon request to the corresponding author.
